# The Immediate Metabolomic Effects of Whole Genome Duplication in the Greater Duckweed *Spirodela polyrhiza*

**DOI:** 10.1002/ajb2.16383

**Published:** 2024-08-01

**Authors:** Tian Wu, Quinten Bafort, Frederik Mortier, Fabricio Almeida-Silva, Annelore Natran, Yves Van de Peer

**Affiliations:** 1 Department of Plant Biotechnology and Bioinformatics, Ghent University, Ghent 9000, Belgium; 2 VIB Center for Plant Systems Biology, VIB, 9052 Ghent, Belgium; 3 Department of Biology, Ghent University, 9000 Ghent, Belgium; 4 College of Horticulture, Academy for Advanced Interdisciplinary Studies, Nanjing Agricultural University Biochemistry, Nanjing 210095, China; 5 Centre for Microbial Ecology and Genomics, Department of Biochemistry, Genetics and Microbiology, University of Pretoria, Pretoria 0028, South Africa

**Keywords:** comparative metabolomics, dosage effects, duckweed, polyploidy, *Spirodela polyrhiza*, whole genome duplication (WGD)

## Abstract

**Premise of the study:**

In plants, whole genome duplication (WGD) is a common mutation with profound evolutionary potential. Given the costs associated with a superfluous genome copy, polyploid establishment is enigmatic. However, in the right environment, immediate phenotypic changes following WGD can facilitate establishment. Metabolite abundances are the direct output of the cell’s regulatory network and determine much of the impact of environmental and genetic change on the phenotype. While it is well known that an increase in the bulk amount of genetic material can increase cell size, the impact of gene dosage multiplication on the metabolome remains largely unknown.

**Methods:**

We use untargeted metabolomics on four genetically distinct diploid-neoautotetraploid pairs of the greater duckweed *Spirodela polyrhiza* to investigate how WGD affects metabolite abundances per cell and per biomass.

**Key results:**

Autopolyploidy increases metabolite levels per cell, but there is considerable variation in the response of individual metabolites. However, the impact on metabolite level per biomass is restricted because the increased cell size reduces metabolite concentration per cell. Nevertheless, we detect both quantitative and qualitative effects of WGD on the metabolome. Many effects are strain specific but some are shared by all four strains.

**Conclusions:**

The nature and impact of metabolic change following WGD depend strongly on the genotype. Dosage effects have the potential to alter the plant metabolome both qualitatively and quantitatively but are largely balanced out by the reduction in metabolite concentration due to an increase in cell size.

## Introduction

With 188,938 known plant metabolites in the PMhub database (Tian et al., 2024) and estimates of over 1 million metabolites (Afendi et al., 2012), the phytochemical repertoire is extremely diverse. Metabolite abundances are the direct output of the cell’s regulatory network, and metabolites provide a link between the genotype and complex phenotypes (Afendi et al., 2012). Secondary metabolites perform a myriad of functions in biotic and abiotic interactions, including gamete production; attraction of pollinators, seed dispersers, symbiotic bacteria and fungi; defence against herbivores and pathogens; wound repair; structural support; salt, drought, desiccation, and UV protection. The plant metabolome consists of a conserved set of primary metabolites and hormones that are required for growth, development, and their regulation, as well as a large range of secondary or specialized metabolites, the synthesis of which is often restricted to a small number of species, and suppression of the germination and growth of competitors (Pichersky and Gang, 2000; Weng et al., 2012; Zhang et al., 2021). Additionally, there is some functional overlap between primary metabolites and phytohormones (Erb and Kliebenstein, 2020). The latter are sometimes even regarded as secondary metabolites (Fàbregas and Fernie, 2022).

With such a diverse range of functionalities, metabolomic alterations and novelty have high evolutionary potential. Indeed, the expansion of the number of plant secondary metabolites in the Cretaceous period is thought to have driven the diversity of flowering plants (Zhang et al., 2021). Similarly, the polymerization of phenolic metabolites into lignin was a key innovation enabling the colonization of the terrestrial realm. Lignin is essential for water conductivity and mechanical support and was also a component of the early plant cuticula (Weng & Chapple, 2010; Renault et al., 2017; González-Valenzuela et al., 2023). Additionally, intraspecific variation in secondary metabolite composition facilitates adaptation at shorter timescales (Moore et al., 2014).

Of all mutations altering the plant metabolome, polyploidization is probably the most spectacular. Although modifications in existing enzymes play a role, gene duplication and, by extension, genome duplication followed by sub and neofunctionalization of enzyme-coding genes lies at the very basis of plant metabolic diversity (Weng et al., 2012; Pichersky & Gang, 2000; Zhou & Liu, 2022). Whole genome duplication (WGD) is a relatively common phenomenon in plant evolutionary history (Wood et al., 2009; Van de Peer et al., 2021) and is much more than a mass manifestation of gene duplication. The duplication of entire batteries of interacting genes, in combination with functional partitioning, functional divergence, gene losses, concerted divergence (Blanc and Wolfe, 2004; Coate et al., 2020), and differential utilization of certain pathways as a result of dosage-sensitive regulation, provides even greater potential for metabolomic differentiation (Gaynor et al., 2020).

Long-term effects of WGD on metabolism are at least partially understood through case studies such as glucosinolate evolution in the Brassicales (Edger et al., 2015), fermentation in *Saccharomyces cerevisiae* (Escalera-Fanjul et al., 2019), and terpenoid biosynthesis in *Lavandula* (Li et al., 2021). However, the immediate effects of polyploidy on the phytochemical spectrum are poorly understood. Nevertheless, chemosystematics was historically one of the most prominent ways to study polyploids, resulting in a rich empirical literature on these immediate effects (Mears, 1980; Bohm, 1987). Many of these previous studies did not find considerable differences caused by autopolyploidy. Those that did reported altered metabolite abundances, metabolite loss, and the emergence of new compounds after WGD, which contributed to Levin’s classic argument for the link between polyploidy and novelty in flowering plants (Levin, 1983). Although the popularity of metabolomic research appeared to have declined among polyploidy researchers with the advent of affordable DNA-sequencing techniques, medicinal plant breeders continued to experiment extensively and successfully with artificial WGD to increase phytopharmaceutical yields (Dhawan and Lavania, 1996; Niazian and Nalousi, 2020). Theoretical explanations for immediate WGD-induced metabolomic change are almost exclusively focused on the increase in gene dosage and its almost equally enigmatic downstream effects on the transcriptome (Doyle and Coate, 2019; Gaynor et al., 2020). Because the proper assessment of dosage effects requires per cell normalization (Coate and Doyle, 2010, 2015), it is hard to reconcile theoretical expectations with empirical data normalized per dry mass. Bridging this gap between theory and observations will lead to a better understanding of the immediate phytochemical effects of WGD.

WGDs raise serious challenges, e.g., minority cytotype exclusion, higher resource demands, and cell cycle problems (Comai, 2005; Levin, 1975; Mortier et al., 2024). However, WGD can also cause immediate changes in phytochemical composition that alter biotic (e.g., pollinator attraction, herbivore deterrence) and abiotic (e.g., drought resistance) interactions (Van de Peer et al., 2021; Fox et al., 2020). As such, they have the potential to overcome the challenges to WGD and facilitate polyploid establishment, a prerequisite for the long-term effects of WGD and diploidization (discussed above) and a central theme in current polyploidy research.

Here, we use discovery metabolomics to investigate the immediate phytochemical effects of autopolyploidy on our model system, the greater duckweed *Spirodela polyrhiza*, a small aquatic macrophyte from the family Lemnaceae. We compare the metabolite abundances in four genetically distinct diploid-autotetraploid pairs to address the potential implications for establishment and combine traditional dry mass normalization with nuclear density information to investigate the link with gene dosage.

## Materials and Methods

### Discovery metabolomics

To get a comprehensive picture of the impact of WGD on the metabolome of *Spirodela polyrhiza*, we opted for discovery or untargeted metabolomics. In contrast to targeted metabolomics, focusing on well-defined groups of metabolites, discovery or untargeted metabolomics aims to detect and quantify as many known and unknown metabolites as possible. To this end, we combined gas chromatography and liquid chromatography with mass spectrometry (GC-MS and LC-MS). Although there is some overlap, GC-MS typically allows the detection of volatile compounds with low molecular weight and low polarity, whereas LC-MS allows the detection of larger, involatile, thermally unstable, polar, and ionic compounds, which make up the bulk of the known plant metabolites. For the LC-MS, we used electrospray ionization in both positive (ESI+) and negative (ESI-) mode. This again increases the number of compounds that can be detected because some compounds ionize exclusively in the positive and some exclusively in the negative mode, but again there is quite some overlap. Due to this overlap, the output of our GC-MS, LC-MS ESI+, and LC-MS ESI- was analyzed separately.

### Spirodela polyrhiza materials

To study the immediate effects of genome doubling in the absence of the confounding effects of both genome merging (causing increased heterozygosity and other hybridization effects) and evolution subsequent to WGD, we used diploids and stable neocolchitetraploids of the model system *Spirodela polyrhiza* (Anneberg et al., 2023a, b; Bafort et al., 2023; Wu et al., 2023; Turcotte et al., 2024). The details of the exact materials we used have been described elsewhere (Bafort et al., 2023). Briefly, four diploid strains of the greater duckweed *Spirodela polyrhiza* (Landolt No. 0013, 9242, 9316, and 9346) were acquired from the Landolt collection in Zurich, Switzerland (recently moved to IBBA-CNR, Milan, Italy). Each of these strains represents a clonal lineage and is affiliated with a distinct population genetic cluster (Xu et al., 2019). Artificial neotetraploids of these four diploid strains were created using colchicine treatment following Wu et al. (2023), and the stability of these lines was ascertained by flow cytometric monitoring for a minimum of two months after recovery.

We started a clonal culture from each of the four diploid and corresponding autotetraploid strains, beginning with a single frond to ensure genetic homogeneity. The plants were grown in Hoagland’s E-medium (Cross, 2002), on temperature-controlled shelves at 24 °C, under a 16/8 light/dark regime at 40 - 45 μmol m^-2^ s^-1^ Photosynthetic Photon Flux Density. Once these cultures had obtained sufficient biomass, eight plants of each culture were transferred to eight random wells spread over eleven six-well plates. After seven days of growth under the aforementioned growth conditions, approximately 50 mg/fresh mass from each well was harvested, patted dry with paper tissue, transferred to pre-weighed microcentrifuge tubes with two 4 mm stainless steel beads, and flash frozen in liquid nitrogen.

### Metabolite extraction

Frozen plant material was ground into powder using a bead beater (MM301, Retsch company, Hann, Germany) at 20 Hz for 1 minute. Following the addition of 1 mL of HPLC-grade methanol (methanol:water = 9:1 v/v), the sample was incubated for 30 minutes at room temperature in a thermomixer (1,000 rpm). The resulting mixture was centrifuged at 21,300 × g for 5 minutes. The pellet was dried at 60 °C for dry mass determination, while the supernatant, containing the extracted compounds, was collected and aliquoted into two microcentrifuge tubes. 900 μL was allocated to LC-MS and 100 μL to GC-MS. The extracted compounds in both tubes were dried at 37 °C under reduced pressure using a SpeedVac system and handed over to the VIB Metabolomics Core Ghent for further processing (https://metabolomicscore-gent.sites.vib.be/en). Five quality control (QC) samples were prepared by pooling equal volumes of all samples.

For LC-MS analyses, the dried methanol fraction was redissolved in 150 μL cyclohexane and 150 μL Milli-Q water, which was then vortexed and centrifuged at 21,300 × g at room temperature for 5 minutes. Finally, 110 μL of the water phase was filtered and analyzed by reversed phase LC-MS analysis. To prevent photoconversion, light exposure was minimized as much as possible.

For GC-MS analyses, the residue obtained from metabolite extraction was trimethylsilylated using a 100 μL derivatization mixture [N-methyl-N-(trimethylsilyl) trifluoroacetamine:pyridine (5:1)].

### UPLC-MS and GC-MS profiling

Following LC-MS sample preparation, 10 μL of the extract was injected twice on a Waters Acquity Ultra Performance Liquid Chromatography (UPLC) system coupled to a Waters Synapt XS mass spectrometer (Waters Corporation, UK) equipped with an electrospray ionization (ESI) source operating in negative (ESI-) and positive ionization mode (ESI+). Chromatographic separation was performed on an ACQUITY UPLC BEH C18 (2.1 mm by 150 mm, 1.7 μL; Waters Corporation, UK) column maintained at 40 °C. A gradient of two buffers was used for chromatographic separation: buffer A (99/1/0.1, H_2_O/acetonitrile/formic acid, pH 3) and buffer B (99/1/0.1, acetonitrile/H_2_O/formic acid, pH 3). Buffer A starts at 99% A and 1% B, and is then decreased to 50% A over 30 minutes and further decreased to 30% A from 30 to 35 minutes, and finally to 0% A from 35 to 37 minutes. Buffer B is then kept at 100% for 2 minutes. Finally, buffer A is increased again to 99% over 1 minute and this ratio is kept for 5 minutes. The flow rate was set to 350 μL/min. All biological samples were analyzed randomly, and pooled samples were included for system reproducibility. Maximum fragmentation coverage was achieved using all ion fragmentation (MSe). For negative and positive ionization, the capillary voltage, sampling cone, and extraction cone settings were set to -2.5 kV, -37 V, and -3.5 V and 2.5 kV, 40 V, and 3.5 V, respectively. The source and desolvation temperatures in both modes were maintained at 120 and 400 °C. The cone gas flow rate was set to 50 L/h in both ionization modes, whereas the desolvation gas flow rates were set to 550 L/h in ESI-mode, and 500 L/h in ESI+ mode. In full MS analysis, collision energy values fluctuated between 4 and 3 V in negative ionization mode, and between 6 and 4 V in positive mode. For data-dependent acquisition MS/MS analysis (DDA MS/MS), an energy ramping approach was implemented, ranging from 10 to 20 eV for low-mass ions and 20 to 45 eV for high-mass ions.

GC-MS analyses were performed with an Agilent 7890B GC system coupled to a 7250 GC/QTOF mass detector (Agilent, Santa Clara, USA). A 1 μL aliquot was injected in splitless mode onto a VF-5ms capillary column (30m × 0.25 mm × 0.25 μm; Varian CP9013; Agilent, Santa Clara, USA) at a constant helium flow rate of 1.2 mL/min. After injection, the oven was held at 80 °C for 1 min, ramped to 280 °C at a rate of 5 °C/min, held at 280 °C for 10 min, ramped to 320 °C at a rate of 5 °C/min, then held at 320 °C for 10 min. The MS transfer line, the MS ion source, and the quadrupole were set to 280 °C, 230 °C, and 150 °C, respectively. The MS detector was operated in EI mode at 70 eV. Full EI-MS spectra were generated for each sample by scanning the m/z range of 50 to 800 with a solvent delay of 7.8 min. All biological samples were analyzed randomly, and pooled samples were included for system reproducibility.

### LC-MS and GC-MS data processing

LC-MS chromatographic data was processed using Progenesis QI 2.4 (Waters Corporation, UK). Raw data was imported with a filter strength parameter of 1. Pooled samples were used for data alignment, system reproducibility, and DDA MS/MS acquisition. Peak detection was based on the results of all analytical runs, and sensitivity settings were set to ‘maximum’ (value = 5). Normalization was done by sample dry mass (Morreel et al., 2006). All detected features, in both negative and positive ionization modes, were aligned across the chromatograms. GC-MS peak detection and alignment were performed using the Profinder software (Agilent, Santa Clara, USA), using a minimum peak height of 300 counts and a minimum ion count of 5 peaks for feature extraction. For alignment, a retention time tolerance of 0.05 min and a minimum dot product of 0.8 was used. After alignment, only compounds with a minimum match factor evaluation (MFE) score of 0.7 in at least two replicates of a given sample group were retained.

### Putative metabolite annotation

Structural annotation of the profiled LC-MS features was attempted by using MS-FINDER v3.56 (Tsugawa et al., 2016). MassBank, GNPS, ReSpect, and an in-house database, PhytoFrag were used for spectral matching. The following databases were used for formula prediction and structural elucidation by *in silico* fragmentation: LipidMAPS, DrugBank, FoodDB, PlantCyc, chEBI, T3DB, NPA, NANPDB, COCONUT, KNApSAcK, PubChem, UNPD, and an in-house database, PhytoComp. The following parameter settings were applied: precursor mass tolerance: 10 ppm; fragment mass tolerance: 10 ppm; and element selection: C, H, N, O, P, and S. Structural matches with a minimum match score of 6 were retained.

For GC-MS, the chromatograms were deconvoluted using Masshunter Unknown Analysis software (Agilent, Santa Clara, USA), and the metabolites corresponding to the deconvoluted spectra were matched against the NIST20 mass spectral library to allow tentative identification. Spectral matches with a minimum match score of 80 were retained.

### Data filtering and normalization

A large portion of the features detected by untargeted metabolomics are uninformative (Mahieu and Patti, 2017). We tried to filter out these uninformative features to increase the power of the downstream analysis (Hackstadt and Hess, 2009). More specifically, we first removed lowly abundant features by exclusively retaining features that were present in all eight replicate samples of at least one specific strain-ploidy combination. Second, we discarded the features with low repeatability by removing the features with the highest (20%) within-group relative standard deviations (RSD, computed as RSD = SD/mean). Finally, the features with the lowest 20% interquartile ranges were removed to get rid of the most invariable features. After filtering, we normalized the data using log2(x+1) transformation. For all filtering steps, we excluded the pooled QC samples. Filtering and subsequent statistical analyses were conducted in the R software suite (version 4.2.3, R Core Team, 2023), unless otherwise stated.

### The dry mass normalized metabolome

To analyze and visualize the relationship between dry mass normalized metabolome and the strain and ploidy level of the samples, we conducted hierarchical cluster analysis (HCA) based on the Spearman correlation between the samples and principal component analysis (PCA) using the *plot_heatmap()* and *plot_PCA()* functions from the BioNERO package (Almeida-Silva and Venancio, 2022).

To quantify how much of the observed metabolomic variation in the filtered feature set can be explained by the genetic background of the strain and how much by the ploidy level, we partitioned the variation (VPA) by means of constrained ordination (RDA, Borcard et al., 1992), and supplemented this with permutational analysis of variance (PERMANOVA, Anderson, 2001), using 999 permutations of the Bray-Curtis dissimilarities (Bray and Curtis, 1957) between samples. For both methods, we used the implementation in the vegan package (Oksanen et al., 2022). Additionally, we quantified the number of features with significant strain, ploidy, and interaction effects using the two-way ANOVA in MetaboanalystR (Pang et al., 2021) with Benjamini & Hochberg correction (Benjamini and Hochberg, 1995) for multiple comparisons.

To determine which features were affected most by WGD, we performed differential abundance analysis in limma (Ritchie et al., 2015), using a general and four strain-specific diploid-tetraploid contrasts. To identify the differentially abundant features (DAFs) that contribute most to the WGD-induced differences, we used partial least-squares discriminant analysis (PLS-DA) implemented in the ropls package (Thévenot et al., 2015). All features with an adjusted p-value < 0.05 and a variable importance in the projection (VIP) exceeding 1 in the successful PLS-DA models were retained.

To interpret the observed metabolomic differences between the strains and the potentially strain-specific effects of WGD in a genetic framework, we determined the genetic distance between the four strains of *Spirodela polyrhiza*. We retrieved the four genotypes’ whole genome resequencing data from Xu et al. (2019) and used BWA-MEM (v0.7.17) (Li and Durbin, 2009) with default parameters to map the reads to the *Spirodela polyrhiza* reference genome (Hoang et al., 2018). We removed duplicates using Picard Tools (v2.6.0) (Broad Institute, 2019) and called SNPs using GATK (v4.1.2) (DePristo et al., 2011). A total of 1,302,676 raw SNPs were identified. The bi-allelic variants were selected and filtered using the following parameters: QD < 5.0, QUAL < 30, FS > 60.0, MQ < 40.0, MQRankSum < -12.5, ReadPosRankSum < -8.0, SOR > 3.0, and 3 > DP > 80. Finally, SNPs with a minor allele frequency below 0.05 were excluded, and we used SnpEff (DePristo et al., 2011) to reduce the dataset further to 6,434 SNPs at synonymous sites. Pairwise genetic distances were calculated with the Tamura-Nei model (Tamura and Nei, 1993) and used to construct a neighbor-joining (NJ) (Saitou and Nei, 1987) phylogenetic tree with 1,000 bootstrap samples (Felsenstein, 1985), using the MEGA software (Tamura et al., 2021). The NJ tree was subsequently visualized using FigTree v1.4.4 (http://tree.bio.ed.ac.uk/software/figtree/).

### Quantifying dosage effects

As increased cell size is one of the most consistent effects of WGD (Bomblies, 2020), while the number of cells per unit of biomass typically differs between cytotypes. Therefore, quantification of the effects of gene dosage on metabolite abundances should rely on metabolite abundances that are normalized per cell and not per biomass. We can infer the tetraploid to diploid ratio of metabolite abundance per cell from information on the sample dry mass and the relative cellular density of tetraploids to diploids (*rcd*) per unit dry mass. For each of the four strains, we estimated *rcd* using flow cytometry on eight replicates of mixed-ploidy samples consisting of approximately 200 mg of fresh mass from each strain. The exact cytotypic composition of the samples was recorded and used in the subsequent analysis. After nuclei extraction and flow cytometry following Wu et al. (2023), we used FlowJo ™ v10.8 Software (BD, New Jersey, USA) to filter out multi-nuclei particles and to select and count the number of diploid and tetraploid nuclei in each sample. To correct for endopolyploidy and cells in the G2 phase, we estimated the proportion of cells with twice the organismal ploidy level (*p*_*G2*_) by performing flow cytometry on five replicates of diploid samples for each strain. To infer the *rcd* and *p*_*G2*_ simultaneously from our mixed-ploidy and diploid samples, we modeled the logit (log-odds) of the proportion of tetraploid nuclei (*p*_*4n*_), as a response variable with a normal error distribution, depending on the log of a linear effects model with the relative mass of the tetraploid in the sample compared to that of the diploid (*rmass* = *mass*_4*n*_*/mass*_2*n*_). Below, we show that the estimated slope corresponds to the relative cell density (*rcd*) and the intercept to the number of endoduplicated and G2 nuclei relative to the number of nuclei in the G1 peak (*p*_*G*2_*/*1 − *p*_*G*2_).

$$\begin{array}{c} logit\left(p_{4n,i}\right)\sim Normal(\mu ,\theta )\\ \mu =\ln\left(rcd_{\left[strain\;\right]}*rmass_{i}+\frac{p_{G2[\;strain\;]}}{1-p_{G2[\;strain\;]}}\right) \end{array}$$

To convert the obtained *rcd* per fresh mass to that per dry mass, we estimated the conversion factor (*f*_*dry*_). We collected 12 samples of fresh material with a fresh mass ranging from 28 mg to 909 g for each strain-cytotype combination. They were weighed and subsequently transferred to an oven with air circulation at 60 °C in pre-weighed aluminum foil envelopes. After 5 days, the samples were weighed on a microbalance within 1 minute of leaving the oven to limit the uptake of air moisture of the sample. We modelled the linear response of dry mass to fresh mass with a normal error distribution, using slope *f*_*dry*_ and no intercept.

$$\begin{array}{c} dm\sim Normal(\mu ,\theta )\\ \mu =f_{dry}*m \end{array}$$

We calculated the relative conversion factor of dry weight of tetraploids compared to diploids (*rf*_*dry*_ = *f*_*dry*,4*n*_*/f*_*dry*,2*n*_) from posterior draws of *f*_*dry*_. Afterwards, we calculated a posterior distribution of relative cell density for dry mass (*rcd*_*dm*_ = *rcd/rf*_*dry*_) from each combination of 500 draws of the *rcd* posterior from model 1 and *rf*_*dry*_ from model 2.

Analysis of the two statistical models was performed using the *brms* package (Bürkner, 2018) for inferring Bayesian linear models and tidyverse package (Wickham et al., 2019). We used Hamiltonian Monte Carlo (HMC) models with two chains, each with 2,000 iterations of which 1,000 were warm-up. We used priors that are weakly regularized by choosing relatively wide prior distributions. We evaluated the performance of the fitted models based on mixing and stationarity in the HMC chains and by checking the effective sample size and $\hat{R}$ statistic for each parameter (McElreath, 2020). Models and priors are further discussed in Appendix S1 (see the Supplementary Data with this article).

To obtain the strain-specific distribution of the tetraploid to diploid ratio of feature abundances, we went back to the unfiltered data and filtered out all features that were absent in at least one of the samples of the studied diploid-tetraploid pairs. As metabolite abundance data is log normally distributed, we log2 transformed all abundances and estimated the log fold change as implemented in the limma package (Ritchie et al., 2015). Log fold changes were transformed into fold change and divided by the mean of estimated strain-specific *rcd*_*dm*_ values to convert them to the per cell normalized values.

### Microscopy

To determine the effect of genome duplication on the cell size of epidermal pavement cells, material was prepared following Bafort et al. (2023) and photographed using an Olympus (Tokyo, Japan) BX51 microscope equipped with an Olympus UplanFLN 40x/0.75na and UplanFLN 20x/0.50na objective. The surface area of epidermal cells was determined in ImageJ (Schindelin et al., 2012).

## Results

### Raw data filtering and annotation

We detected 10,042 ‘features’, representing chemical compounds, using LC-MS ESI-, 7,604 features using LC-MS ESI+, and 132 features using GC-MS. After the first, second, and third filtering steps, these numbers were reduced to 7,794, 6,235, and 4,988 for the LC-MS ESI-; to 4,978, 3,982, and 3,186 for the LC-MS ESI+; and to 107, 85, and 68 for the GC-MS, respectively. Only a small portion of these, i.e., 80 LC-MS ESI-features, 17 LC-MS ESI+features, and 58 GC-MS features, could be given a putative annotation. Detailed information about the identified metabolites can be found in Appendix S2, Table S1 (for LC-MS ESI-), Table S2 (for LC-MS ESI+), and Table S3 (for GC-MS).

### The effects of strain and ploidy level on the metabolome

**Figure 1:**
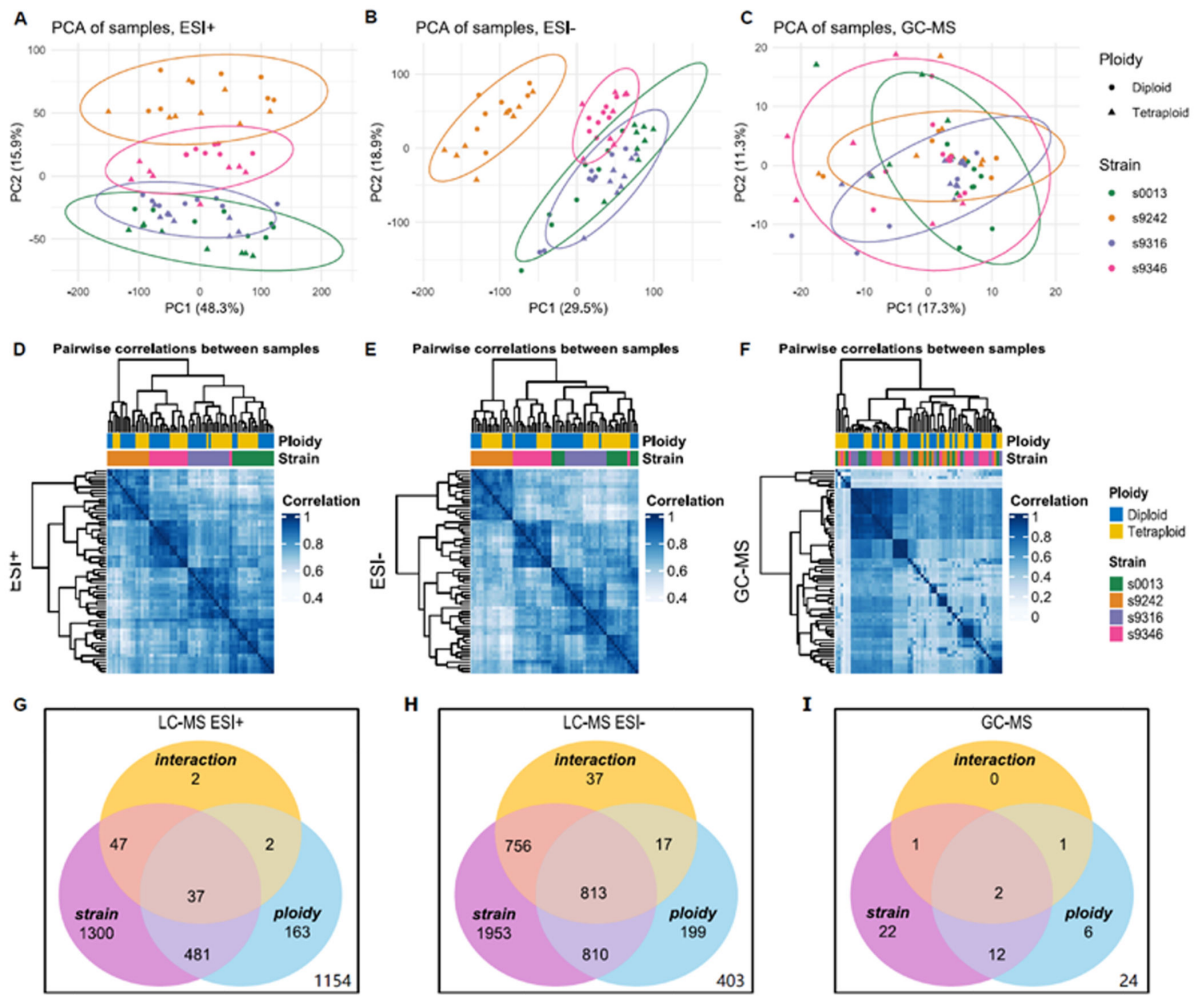
Metabolite profile analysis for four diploid-autotetraploid pairs of *Spirodela polyrhiza* using principal component analysis, hierarchical cluster analysis, and two-way ANOVA for feature sets detected using LC-MS ESI+, LC-MS ESI-, and GC-MS. PCA score plots (A, B, C) visualize overall metabolite profiles for all samples, with shaded ellipses indicating the 95% confidence area around each cluster, strains are color-coded, diploids are represented by dots, tetraploids by triangles. Clustered heatmaps (D, E, F) of the Spearman correlations amongst the metabolite profiles of the different samples in ESI+, ESI-, and GC-MS, the genetic background and ploidy level of the samples is indicated as color (diploid as blue, tetraploid as orange) in the horizontal bars above the heatmaps. Venn diagrams (G, H, I) provide an overview of the number of features with significant (padj < 0.05) ploidy, strain, and interaction effects.

**Figure 2:**
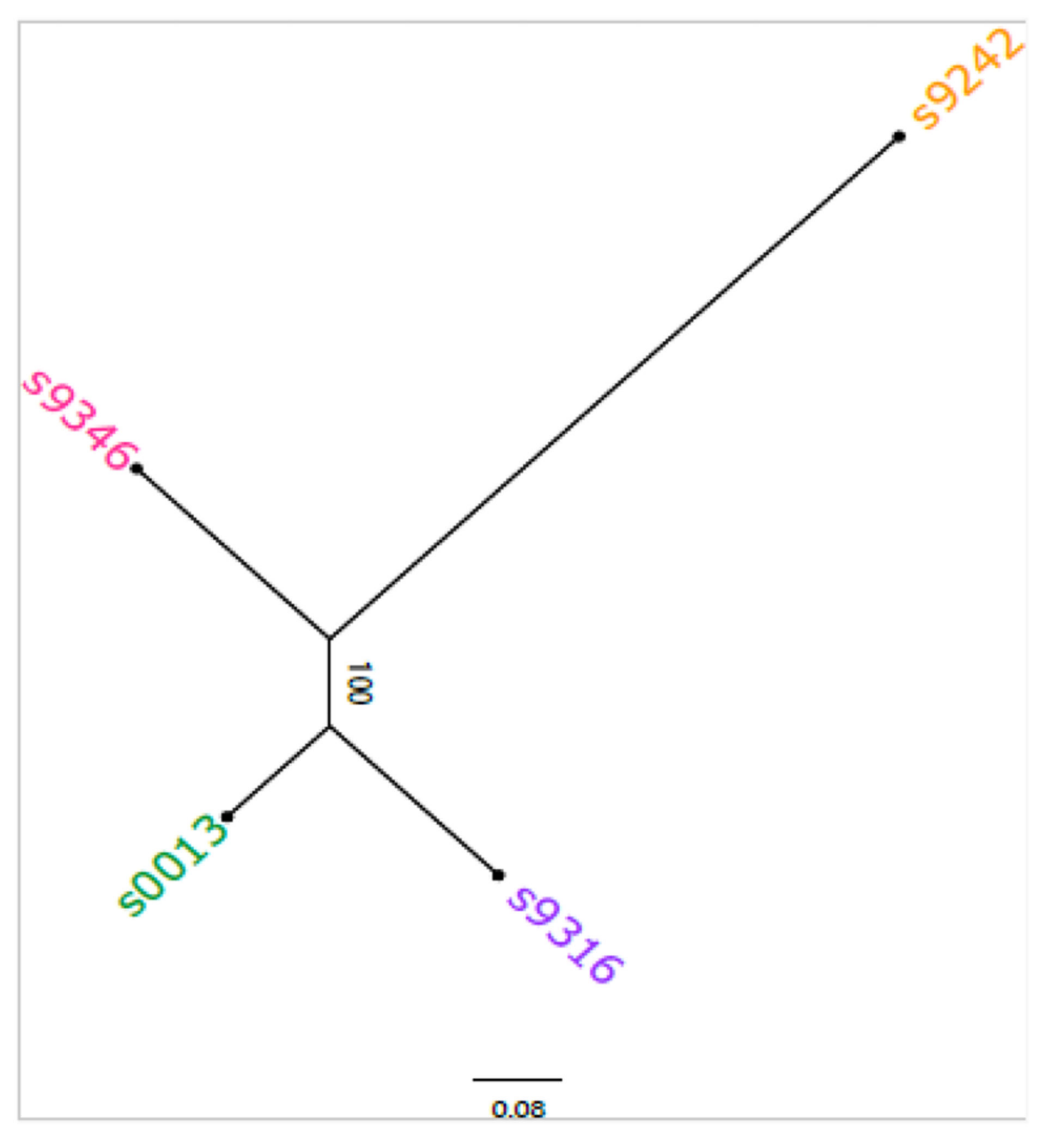
A neighbor-joining tree depicting the genetic relationships among the four clonal *Spirodela polyrhiza* strains based on the pairwise genetic distances using SNPs at synonymous sites.

Variation partitioning analysis (VPA, Table 1) and PERMANOVA (Table 2) indicate large differences in the proportions of the explained variation between all three datasets. The VPA results (Table 1) indicate that only half of the variation for LC-MS ESI-, one-third of the variation for LC-MS ESI+, and almost none of the variation in the GC-MS dataset can be explained by ploidy level and strain. Nevertheless, for all three datasets, most of the explained sample variation can be attributed to strain effects, with polyploidy making a smaller but significant contribution. The permutational multivariate analysis of variance (PERMANOVA; Table 2) further indicates that the interaction between strain and ploidy level has a small but significant contribution to the variation in the features detected by LC-MS ESI- and GC-MS. Both the PCAs (Fig. 1A, 1B, 1C), and the hierarchical clustering analysis (Fig. 1D, 1E, 1F), corroborate these results. For both LC-MS feature sets, the samples cluster primarily by strain, followed by a ploidy-related sub-clustering. However, there are two deviations from this pattern. First, for the LC-MS ESI-clustering (Fig. 1E), most of the diploid and tetraploid samples of strains 0013 and 9316 appear to cluster together and are only secondarily split by their genetic background. The same pattern can be observed in the Spearman correlation heatmaps (Fig. 1E). Second, in the LC-MS ESI+ hierarchical clustering analysis (Fig. 1D), most of the tetraploid samples of strain 9242 cluster with the samples of strain 9346. However, the corresponding heatmap shows that these tetraploids have a larger affinity with the diploid and the remaining tetraploid samples of their own strain (Fig. 1D). Strikingly, the tight clustering of 0013 and 9316, and their clustering closer to 9346 than to 9242 reflects the genetic relationships of these strains (Fig. 2). The primary ploidy-related clustering of 0013 and 9316 in the LC-MS ESI-analysis seems to suggest that there is not only a phylogenetic pattern in the metabolome, but that the effects of WGD are also more similar for strains that share a more recent ancestry despite high within-strain variation.

The feature-specific ANOVA results (Fig. 1G, 1H, 1I) indicate that for both filtered LC-MS datasets, about 40% of the features are significantly affected by strain alone, 4-5% exclusively by ploidy level, and 15% by the combination of both without interaction. For the LC-MS ESI-dataset (Fig. 1H), there are an additional 32% of the features with a significant interaction effect, while the significance of the interaction between ploidy and strain is restricted to 2.8% of the features in the LC-MS ESI+ dataset (Fig. 1G). Approximately 65% of the features in the filtered GC-MS dataset (Fig. 1I) have either a significant strain, ploidy, or interaction term. Of these features, 32% have only a significant strain effect, 9% only a significant ploidy effect, 18% have both a significant ploidy and a significant strain effect without any interaction effect, and 6% have a significant interaction term.

### WGD induced novelty and differential abundances

**Figure 3:**
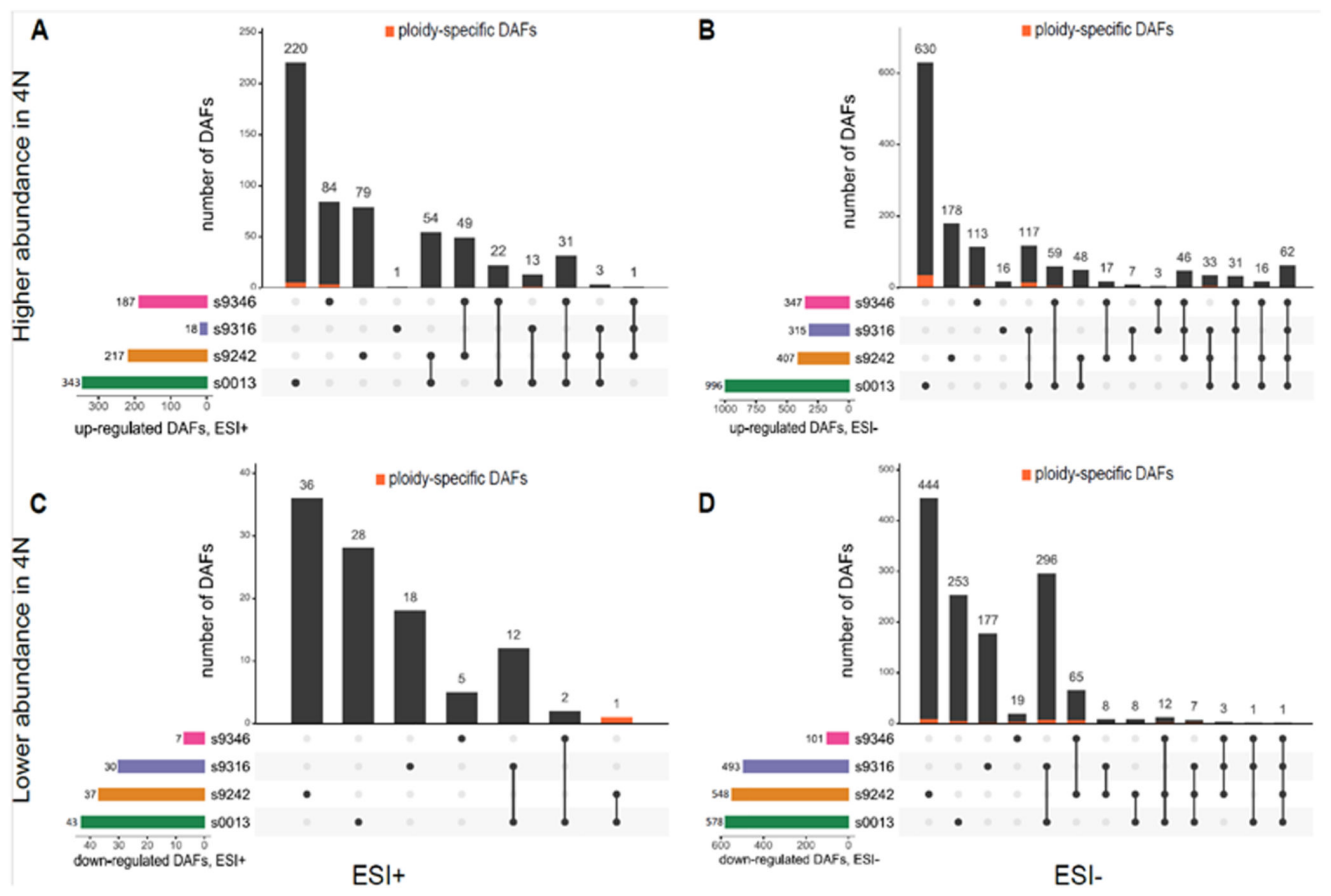
UpSet plots of differentially abundant features (DAFs) in four *Spirodela polyrhiza* strains detected using the two LC-MS modes: ESI+ and ESI-. For the corresponding plots visualizing the few DAFs in the GC-MS feature set, see Appendix S1, Fig 1. The horizontal bar plots represent the total number of DAFs for each strain and reveal that strain 0013 is by far the richest in DAFs. The vertical bar plots indicate what proportion of these DAFs is unique to each strain (single dots) and how shared DAFs are spread over the different strain combinations (black dots connected by a vertical black line). The ploidy-specific DAFs, present in one but absent in the other ploidy level, are indicated in orange. Panels A and B display features with significantly higher abundances in the tetraploids for ESI+ and ESI-mode respectively. Panels C and D represent the features with significantly lower abundances in the tetraploids in ESI+ mode (C) and ESI-mode (D).

In total, we detected 2,542 LC-MS ESI-, 636 LC-MS ESI+, and 36 GC-MS features with significantly different abundances (DAFs) per biomass between tetraploids and diploids, in at least one of the strains. The number of DAFs per strain (the horizontal bar plots in Fig. 3) cover only a fraction of all detected features. Strain 0013 has the most DAFs, covering only 32% and 12.5% for the LC-MS ESI- and ESI+ features sets, respectively (the horizontal bar plots in Fig. 3). These DAFs reflect mainly quantitative but also some qualitative changes after WGD. 90 LC-MS ESI- and 10 LC-MS ESI+ DAFs are ploidy-specific in at least one of the strains (Fig. 3, Appendix S2, Table S4 and Table S5), and are either present in the tetraploids and absent in the diploids (57 in the ESI- and 9 in the ESI+) of a specific strain, or the other way around (33 in the ESI- and 1 in the ESI+). Higher abundance, lower abundance, and ploidy-specific DAFs are found in all strains. Many features show similar responses to WGD over multiple strains, but some responses are unique to a single strain (Fig. 3, Appendix S1, Fig. S1 for GC-MS). In general, there is little congruence in the DAFs over the different strains. The significance of the WGD-induced increase or decrease in metabolite abundance is confined to a single strain for at least 68% of the DAFs in five of the six DAFs data sets (Fig. 3, Appendix S1, Fig. S1). The five GC-MS features with a higher abundance in the tetraploids are the only exception (Appendix S1, Fig. S1). In sharp contrast with these results, only 63 DAFs, all identified in the ESI-mode, are shared by all four strains, indicating a consistent effect of WGD over the four strains. For 62 of these 63 features, abundances are higher in tetraploids, while only one shows higher abundances in diploids (Fig. 3B, 3D). Overall, there are more features with a higher abundance in tetraploids, but there are large differences between the strains. For both LC-MS analyses, strains 0013 and 9346 have more features with higher abundance in tetraploids than in diploids, whereas strain 9316 has less (Fig 3, Appendix S2, Table S6). Strain 9242 has more in the ESI+ analysis and less in the ESI-analysis (Fig 3, Appendix S2, Table S6).

Although no DAFs relating to qualitative changes within a strain were annotated, we were able to annotate 35 DAFs in the LC-MS ESI-, 3 in the LC-MS ESI+, and 21 in the GC-MS. An overview of these annotations can be found in Appendix S2, Table S7 (for LC-MS ESI-), Table S8 (for LC-MS ESI+), and Table S9 (for GC-MS). Among these annotations, citric acid (LC-MS ESI- in Appendix S2, Table S7) and fructofuranose (GC-MS in Appendix S2, Table S9) are the only metabolites showing a consistent increase in abundance across all tetraploid strains in comparison to diploids.

### Dosage effects

**Figure 4:**
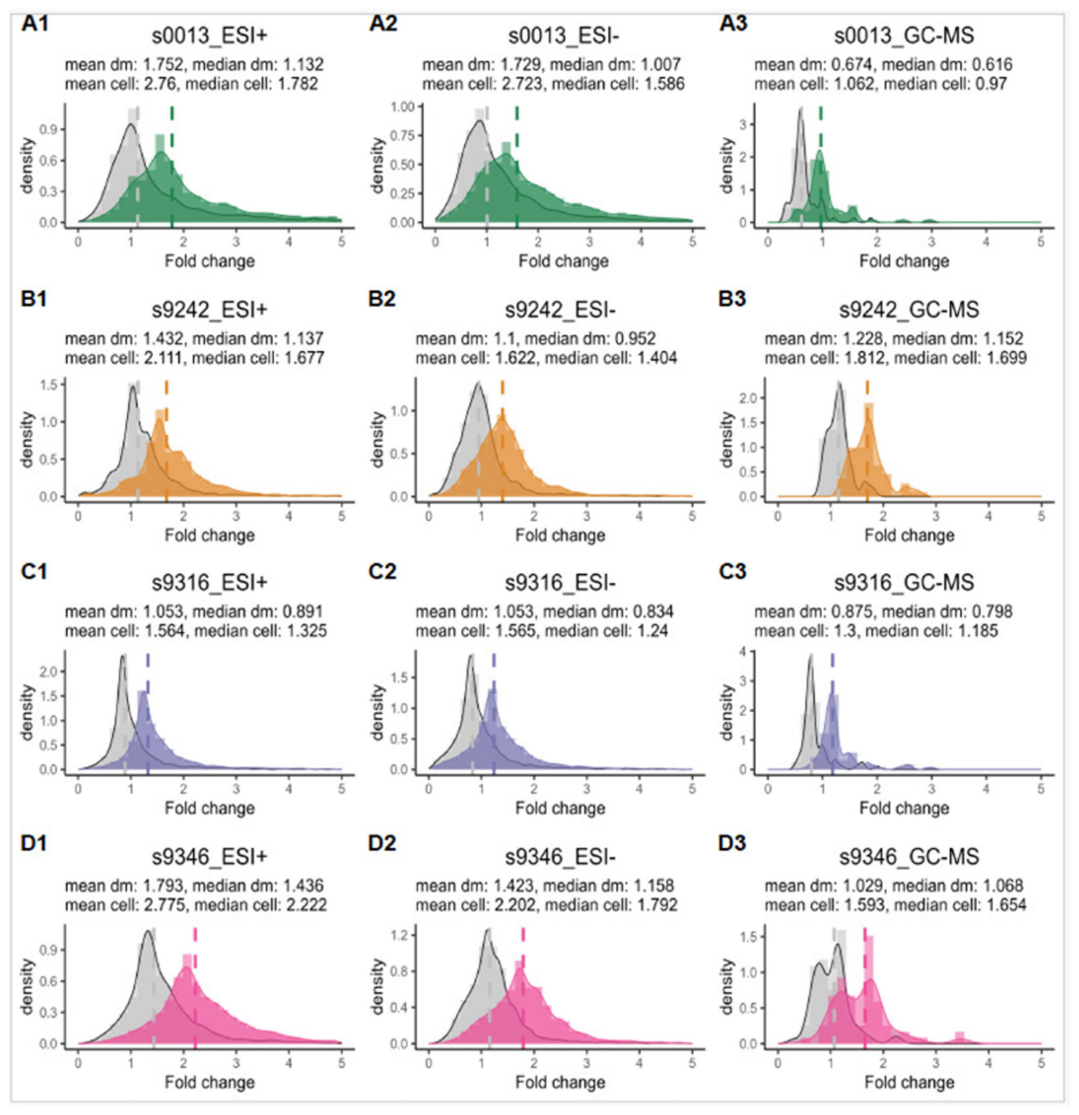
The distributions of the tetraploid to diploid ratio of metabolite abundances per dry mass (FCdm, in grey) and per cell (FCcell, in color) for all four *Spirodela polyrhiza* strains across three modes: LC-MS ESI+, LC-MS ESI-, and GC-MS, visualized by histograms and kernel density plots. The x-axis represents the tetraploid to diploid ratio (fold change), while the y-axis gives the probability density function for the kernel density estimator. The dashed vertical lines indict the median fold change values of FCdm and FCcell.

Overall, the distributions of the tetraploid to diploid ratio of metabolite abundances per dry mass (fold change, FCdm) are wide and right skewed, ranging from the near absence in the tetraploids (FC = 0) to more than a five-fold increase in metabolite levels (Fig. 4). Nevertheless, when ignoring the GC-MS feature set of strain 0013 (median FCdm = 0.616, see Fig. 4A3), and the LC-MS ESI+ feature set of strain 9346 (median FCdm = 1.436, see Fig. 4D1), the distributions are generally centered around the no-change value (i.e., 1, see median FCdm in Fig. 4). This is well in line with the results of the variance partitioning and PERMANOVA assigning little importance to ploidy effects, and the restricted number of features with significantly altered abundances per strain.

**Figure 5:**
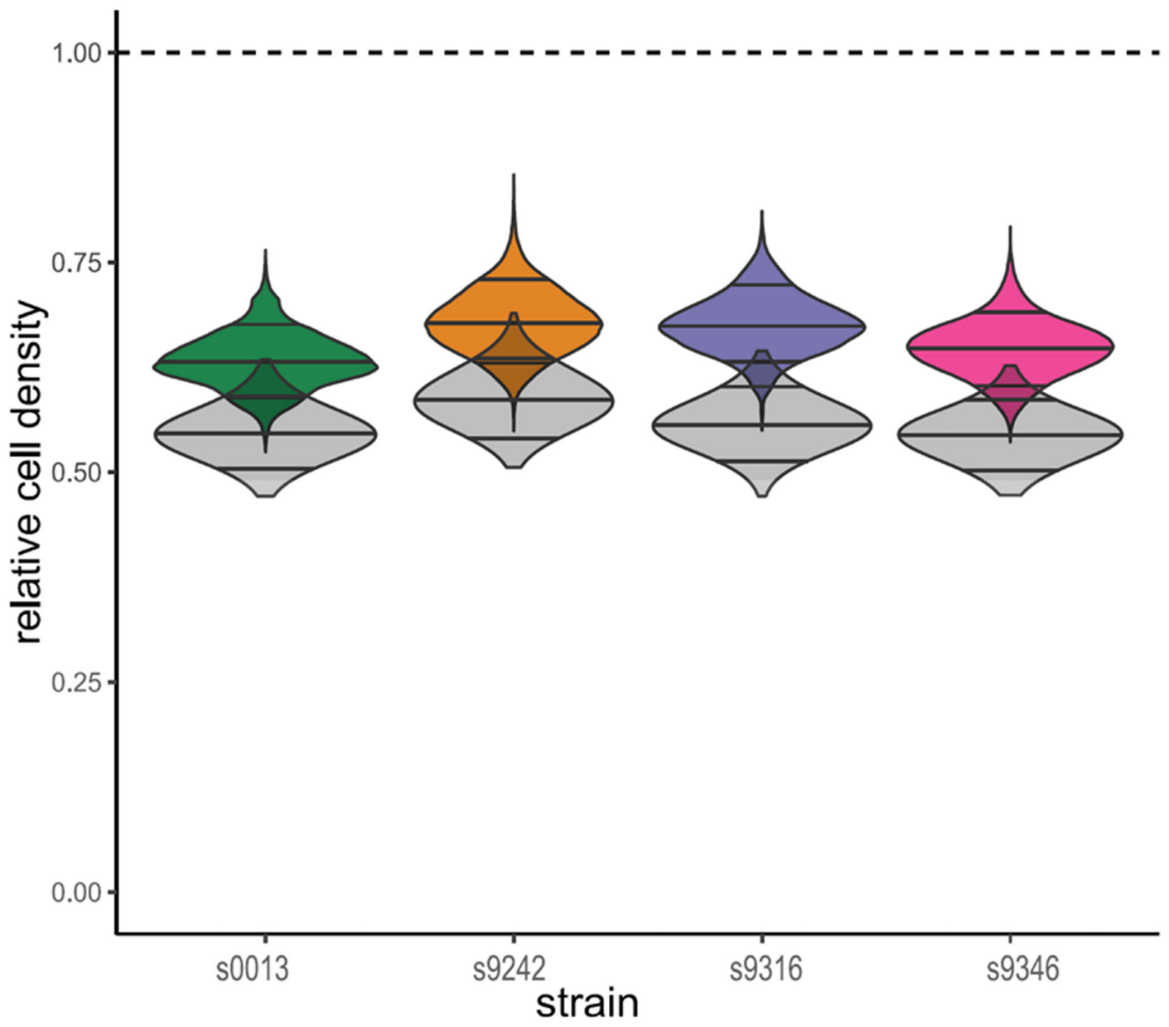
Posterior distributions of the strain-specific relative cell density standardized for dry mass (rcddm, in color) and fresh mass (rcdfm, in grey) with 0.09, 0.5, and 0.91 percentiles indicated. The dashed line indicates the value if tetraploid tissue had equal cell density compared to diploids.

The mixed-cytotype flow cytometric analysis indicates that the diploids of the four studied strains have almost twice as many cells per unit fresh mass as the tetraploids (Appendix S1, Fig. S2). Because WGD reduces the dry to fresh mass ratio (see Appendix S1, Fig. S3), the tetraploid to diploid ratio of the number of nuclei per unit dry mass falls slightly higher between 0.6 and 0.7 (Fig. 5). Consequently, when dividing the FCdm distribution by the mean estimated relative cell density (*rcd*_*dm*_) to obtain the tetraploid to diploid ratio of metabolite abundances per cell (FCcell), the distributions shift to the right (Fig. 4). The median of the GC-MS features of strain 0013 (Fig. 4A3) and the median of the LC-MS ESI+ feature set of strain 9346 (Fig. 4D1) becomes 0.97 and 2.222, respectively. And the medians of the other strain-feature set combinations range between 1.185 and 1.783. Because the median FCcell corresponds well to the peaks of the distribution of the FCcell values, the most common response falls between full dosage compensation (FCcell = 1) and a 1:1 dosage effect (FCcell =2), which here tends to conserve the per biomass concentration of metabolites. However, the distributions remain wide, and for all strains there are quite some features showing a negative dosage effect (overcompensation) where the FCcell <1, and positive dosage effects where the FCcell > 2. The overall strength of dosage effects differs between strains. Dosage compensation is most pronounced for strain 9316 (1.185 < median FCcell < 1.325) and less for the others (FCcell > 1.404, when ignoring the GC-MS features of strain 0013).

## Discussion

Building on a long tradition within polyploidy research, we used discovery metabolomics to investigate the immediate effects of WGD on the metabolome of the greater duckweed *Spirodela polyrhiza*.

### WGD increases average metabolite abundance per cell

Ultimately, all immediate effects of autopolyploidy result from an increase in gene dosage and an increase in the bulk amount of DNA, i.e., the nucleotype (Bennett, 1971; Bennett, 1972; Doyle & Coate, 2019). How these two effects at the genome level affect the transcriptome, proteome, and metabolome to ultimately determine the phenotype, is largely unknown. A proper understanding of this “omics” cascade starts with proper normalization (Coate and Doyle, 2010, 2015). While per biomass normalization is valuable to infer phenotypic effects on tissues and individual organisms, per cell or per genome normalization is a necessity to understand the effects of gene dosage on the cell’s phenotype, especially since the nucleotype scales with cell size and the cell density per unit of biomass is bound to change after WGD. To the best of our knowledge, we here provide the first assessment of dosage effects per cell at the metabolome level. We show that, despite much variation in the response of individual metabolites, the average metabolite abundance per cell increases but shows some dosage compensation. Our results are largely in line with simulations and the effects observed at the transcriptome and proteome level. *In silico* simulations of increased gene dosage on gene regulatory networks show that the duplication of biological networks has no qualitative effects on gene expression (Wagner, 1994), while quantitative simulations predict a magnification of gene repression and stimulation, even when assuming full dosage compensation (Ebadi et al., 2023). Empirical data on shifts in gene expression is scarce as it requires rarely applied per cell or per genome normalization. Studies on the effects of WGD on the transcriptome have been restricted to neoautotetraploid *Zea mays* (Guo et al., 1996), the natural allopolyploid *Glycine dolichocarpa* (Coate and Doyle, 2010), the natural autopolyploid *Tolmiea menziesii* (Visger et al., 2019), and synthetic and natural autopolyploids of *Arabidopsis thaliana* (Hou et al., 2018; Song et al., 2020). From these systems we know that an increased gene dosage is often translated into an increased transcription per cell. A 1:1 dosage response is common in autopolyploids but there is much variation in the individual dosage responses among the different genes and there are substantial fractions of the genes that show dosage compensation, negative dosage effects, and more than twice the diploid expression per cell. Information at the proteomic level is only available for *Zea mays* and corresponds well to that at the transcriptomic level (Birchler & Newton, 1981; Yao et al., 2011; Doyle & Coate, 2019). Similarities between what is observed at the ‘metabolome’ and the ‘proteome and transcriptome’ level would suggest that most of the immediate effects can be traced back to the transcriptome and proteome levels and their interactions, but such relations can probably only be studied when the different levels are assessed within the same species. Furthermore, enzyme reaction rates are non-linear and do not depend on the absolute quantities of enzyme and substrate within the cell but rather on their local concentration. Consequently, the effects of increased levels of proteins on the metabolome might be reduced by an increased cell size. If the increased cell size is mainly a nucleotypic effect, this compensating effect could be seen as nucleotypic dosage compensation, maintaining metabolic rates in larger cells.

### Cell size effects balance out dosage effects

The dry mass-normalized metabolome highlights how the abundance within a unit of biomass is conserved for many compounds. While cells may produce and/or store more of a metabolite, tissue, or individuals (with equal biomass) may produce or store that metabolite at equal quantities. Variation in per biomass shift in metabolite abundance remains large but instead of a 1:1 dosage effect, most of the features show no change. The limited power of ploidy level to explain the overall variation in the metabolome confirms that the “per biomass” concentration of few metabolites are affected by the inherent effects of autopolyploidy itself.

### WGD has quantitative and qualitative metabolic effects

Although the overall observed differences in the metabolome per unit of dry mass were rather limited, WGD induced quantitative, and to a lesser extent qualitative, changes in the metabolome. Especially the qualitative differences are interesting, because the absence of such differences between a polyploid and a potential diploid ancestor was historically used as an argument for autopolyploidy (e.g., Soltis & Bohm, 1986; Soltis et al., 1983, Bohm, 1987). Furthermore, a recent meta-analysis by Gaynor et al. (2020) suggests that differences in metabolite concentration and diversity are rarely detected in metabolomic studies on natural polyploids. However, the same authors contrasted the results from these natural populations with cultivated and artificially induced polyploids, like ours, where such effects are more commonly observed (Haskell, 1968; Levy, 1976; Dhawan and Lavania, 1996; Parida and Misra, 2015). These contrasting results could be explained by artificial selection to maintain a higher concentration of a novel metabolite in the cultivars and natural selection for energy efficiency in the natural polyploids (Gaynor et al. 2020).

### Genotype is the main driver of metabolomic variation and modulates the response to WGD

The genetic background of the strain, reflecting its evolutionary history, seems to explain the lion’s share of the explained metabolomic variation. The metabolome of genetically closer strains is more similar than that of more distantly related strain pairs. Geographic variation in plant metabolomes is well-known and thought to reflect local adaptation (Davey et al., 2008; Züst et al., 2012; Meijón et al., 2016; Iwanycki Ahlstrand et al., 2018). Although our four strains can be assigned to four distinct genetic clusters with a characteristic geography, ecological conditions within these clusters are very diverse. Because environmental data from the sampling sites is missing, additional research is needed to determine whether this metabolic similarity is the result of genetic similarity, or adaptation to comparable environments.

Despite the dominance of these strain effects on the “per biomass” concentration of metabolites, WGD still has a significant effect on many features. And like other aspects of the phenotype of *Spirodela polyrhiza* (Anneberg et al., 2023b; Anneberg et al., 2023a; Bafort et al., 2023; Turcotte et al., 2024) and that of other plants (Van Drunen and Husband, 2018; Münzbergová and Haisel, 2019; Wei et al., 2020; Pacey et al., 2022), the immediate effect of WGD on the phytochemical composition of *Spirodela polyrhiza* is determined partially by the interaction between the genetic background and the ploidy level, highlighting the importance of multiple origins for polyploid establishment. The presence of DAFs, whether differentially abundant in a single or multiple strains, indicate clearly that WGD has the potential to have an ecological impact, but this will largely depend on the specific metabolites affected, which in turn seems to be genotype specific.

### Assumptions

By working with whole plant material, for both metabolite extraction and mixed flow cytometry, we implicitly assumed that the effects of WGD on cell size and metabolite levels are consistent over all sampled tissues. Since the effects of WGD on cell size are known to differ among cell types in *Arabidopsis* (Katagiri et al., 2016) and *Gossypium* (Snodgrass et al., 2017), our assumption of a uniform response is probably unrealistic. But, because the bulk of a *Spirodela polyrhiza* plant consists of parenchymatic cells, which show little differentiation (Landolt and Kandeler, 1987), the observed quantitative differences should largely reflect changes in the parenchyma. Nevertheless, compounds that show extreme responses in non-parenchymatic tissues might increase the variation in the effects of gene dosage. The effect of WGD on the size of parenchymatic cells has never been assessed in *Spirodela polyrhiza*. But the low observed tetraploid to diploid ratio of cell densities suggests a similar increase as that of the stomatal guard cells (Bafort et al., 2023) and epidermal pavement cells (Appendix S1, Fig. S4). Additionally, in autotetraploid *Lemna gibba*, an increase in cell size has been reported for different cell types in the frond and the root (Sarin et al., 2023).

To further disentangle the effects of cell size and dosage on cellular phenotypes in the future, information on WGD induced transcriptomic, proteomic, and metabolomic changes should be combined with cell size data. Upcoming technologies as spatial transcriptomics and single cell omics have tremendous potential in this context.

## Conclusion

In summary, our study considered the immediate effects of whole genome duplication (WGD) on *Spirodela polyrhiza*’s metabolome, expanding upon established polyploidy research. WGD induces a cascade of effects, impacting transcriptomic, proteomic, and metabolomic levels. Per-cell normalization reveals considerable variation in metabolic response, but overall, autopolyploidy increases metabolite abundances per cell. Because WGD simultaneously increases the cell size, the per-biomass abundance of many metabolites remains unaltered after WGD. We see this reflected in strain-specific effects dominating the shifts in metabolite abundance “per biomass”. Nevertheless, WGD still exerts a discernible quantitative and qualitative influence on the phytochemical composition of our strains, each with potential ecological implications. Some metabolite levels are consistently higher in all tetraploids but many effects are restricted to a single strain reflecting the importance of the genetic background.

## Supplementary Material

Supplementary Materials and Figures

Supplementary Tables

## Figures and Tables

**Table 1:** The contributions of genetic background (strain) and ploidy level on the variation between *Spirodela polyrhiza* metabolomes quantified using variance partitioning analysis. The fraction explained by strain consist of the variation explained by strain alone (a) and the variation explained by strain and ploidy simultaneously (c). Similarly, the fraction explained by ploidy level consists of the variation explained by ploidy alone (b) and the variation explained by ploidy and strain simultaneously (c).

		ESI+		ESI-		GC-MS	
Source	Df	R.squared	Adj.R.squared	R.squared	Adj.R.squared	R.squared	Adj.R.squared
[a+c] = Strain	3	0.29959	0.26457	0.4476	0.41998	0.07427	0.02799
[b+c] = Ploidy	1	0.05608	0.04085	0.06747	0.05243	0.02396	0.00822
[a+b+c] = Strain + Ploidy	4	0.35566	0.31198	0.51507	0.48219	0.09824	0.0371
Individual fractions							
[a] = Strain|Ploidy	3		0.27113		0.42976		0.02888
[b] = Ploidy|Strain	1		0.04741		0.06222		0.00911
[c]	0		-0.00656		-0.00978		-0.00089
[d] = Residuals			0.68802		0.51781		0.9629

**Table 2:** Results of the permutational multivariate analysis of variance (PERMANOVA) illustrating the effects of the independent variables strain, ploidy level, and their interaction on the metabolome of *Spirodela polyrhiza* (*: p < 0.05, **: p < 0.01, ***: p < 0.001).

		ESI+				ESI-				GC-MS			
Metadata	Df	Sums of squares	Variation (R2)	F.model	Pr (>F)	Sums of squares	Variation (R2)	F.model	Pr (>F)	Sums of squares	Variation (R2)	F.model	Pr (>F)	
strain	3	0.18749	0.26247	7.4884	0.001***	0.17228	0.44438	20.2761	0.001***	0.008089	0.14422	3.7751	0.001***	
ploidy	1	0.03502	0.04902	4.1958	0.012*	0.02585	0.06669	9.1285	0.001***	0.002825	0.05037	3.9553	0.004**	
strain:ploidy	3	0.02447	0.03425	0.9773	0.431	0.03095	0.07982	3.6422	0.001***	0.005176	0.09228	2.4156	0.002**	
Residual	56	0.46737	0.65426			0.1586	0.40911			0.04	0.71313			
Total	63	0.71435	1			0.38768	1			0.056091	1			

## Data Availability

All raw LC-MS and GC-MS files will be deposited in the EMBL-EBI MetaboLights database (DOI: 10.1093/nar/gkad1045, PMID:37971328) with the identifier MTBLS10435, which can be accessed at https://www.ebi.ac.uk/metabolights/MTBLS10435. The codes used for the analyses in this paper will be available on a GitHub repository at https://github.com/wutian1217/metabolomics.
