# Supplementary material for: The Immediate Metabolomic Effects of Whole Genome Duplication in the Greater Duckweed Spirodela polyrhiza
Source: Am J Bot. Author manuscript; Available in PMC 2025 Feb 1. (PMC7616399; doi:10.1002/ajb2.16383)
Supplement: Supplementary Materials and Figures [file EMS198113-supplement-Supplementary_Materials_and_Figures.docx]

**Appendix S1. Supplementary Materials and Figures**

**The Immediate Metabolomic Effect of Whole Genome Duplication in the Greater Duckweed *Spirodela polyrhiza***

Tian Wu^1,2*^ Quinten Bafort^1,2,3*#^, Frederik Mortier^1,2,3^, Fabricio Almeida-Silva^1,2^, Annelore Natran^2^, Yves Van de Peer^1,2,4,5#^

^1^ Department of Plant Biotechnology and Bioinformatics, Ghent University, Ghent 9000, Belgium

^2^ VIB Center for Plant Systems Biology, VIB, 9052 Ghent, Belgium

^3^ Department of Biology, Ghent University, 9000 Ghent, Belgium

^4^ College of Horticulture, Academy for Advanced Interdisciplinary Studies, Nanjing Agricultural University Biochemistry, Nanjing 210095, China

^5^ Department of, Genetics and Microbiology, University of Pretoria, Pretoria 0028, South Africa

This PDF file includes:
Supplementary materials 1-2
Supplementary figures S1 to S4

Supplementary material 1: model derivation

We only consider diploid and tetraploid nuclei. We will derive the relation of proportion of measured tetraploid nuclei ($p_{4n}=\frac{n_{4n}}{n_{2n}+n_{4n}}$) to the mass of diploids ($m_{2n}$) and tetraploids ($m_{4n}$) used in a sample, the relative cell density ($rcd$) and the proportion of cells in G2 ($p_{G2}$) whose nuclei measures double the ploidy as the cell it stems from. We will not start from the proportion of tetraploid nuclei but rather the related odds, i.e., relative number of tetraploid nuclei compared to diploid, for ease of derivation.

The number of nuclei measured of each cytotype by the flow cytometer is assumed to be proportional all nuclei of that cytotype in the sample. The number of diploid nuclei ($n_{2n}$) is determined by the number of diploid cells subtracted with those that have tetraploid nuclei because they are in G2. The number of tetraploid nuclei ($n_{4n}$) is determined by the number of tetraploid cells subtracted with the number of tetraploid cell in G2 but increased with the number of diploid cells that are in G2.

$$\frac{n_{4n}}{n_{2n}}=\frac{c_{4n}-p_{G2}*c_{4n}+p_{G2}*c_{2n}}{c_{2n}-p_{G2}*c_{2n}}$$

With $c_{j}$ indicating the number of cells for cytotype j.

$$\frac{\frac{c_{4n}}{c_{2n}}*\left( 1-p_{G2} \right)+p_{G2}}{1-p_{G2}}$$

$$\frac{c_{4n}}{c_{2n}}+\frac{p_{G2}}{{1-p}_{G2}}$$

The number of cells of each cytotype j in the sample ($c_{j}$) is determined by product of the mass of that cytotype ($m_{j}$) with the cellular density of that cytotype j (${cd}_{j}$, number of cells per unit mass).

$$\frac{m_{4n}*{cd}_{4n}}{m_{2n}*{cd}_{2n}}+\frac{p_{G2}}{{1-p}_{G2}}$$

$$rmass*rcd+\frac{p_{G2}}{{1-p}_{G2}}$$

Because the odds of tetraploid nuclei (${n_{4n}}/{n_{2n}}$)is limited to strictly positive values, we scale this with a natural log function to encompass all rational value on which a Normal error distribution can be modelled.

$$ln\left( \frac{n_{4n}}{n_{2n}} \right)=ln\left( rmass*rcd+\frac{p_{G2}}{{1-p}_{G2}} \right)$$

The left side, the log of an odds, equals the logit of its proportion, which is not coincidentally a common scaling function for proportions.

$$logit\left( p_{4n} \right)=ln\left( rmass*rcd+\frac{p_{G2}}{{1-p}_{G2}} \right)$$

The estimate for cellular density here is done with fresh weight. Also wanted to calculate a cellular density on dry weight (*rcddm*). Because dry mass (dm) is the product of fresh mass and the dry mass conversion factor, we derive rcddm as follows:

$$rcddm=\frac{\frac{c_{4n}}{{dm}_{4n}}}{\frac{c_{2n}}{{dm}_{2n}}}=\frac{\frac{c_{4n}}{m_{4n}*f_{dry,4n}}}{\frac{c_{2n}}{m_{2n}*f_{dry,2n}}}=\frac{rcd*f_{dry,2n}}{f_{dry,4n}}=\frac{rcd}{{rf}_{dry}}$$

Supplementary material 2: full model description

Model 1:

$$logit\left( p_{4n,i} \right) Normal\left( \mu,\theta\right)$$

$$\mu=ln\left( {rcd}_{\left[ strain \right]}*{rmass}_{i}+{rG2}_{\left[ strain \right]} \right)$$

We estimated logit transformed proportions of tetraploid nuclei as the response variable with normal error distribution and a log-link to the linear effect of the sample’s $rmass$. We estimate a strain-specific slope, equal to that strains relative cell density of tetraploids ($rcd$) and a strain-specific relative number of cells in G2 ($rG2=\frac{p_{G2}}{{1-p}_{G2}}$). We used the following priors:

$${rcd}_{\left[ strain \right]} e^{Normal\left( 0,2 \right)}$$

$${rG2}_{\left[ strain \right]} e^{Normal\left( -3,2 \right)}$$

$$\theta Cauchy\left( 0,0.5 \right)$$

We limit the prior of *rcd* and *rG2* to strict positive values by applying the exponential function to a normal distribution because it represents a ratio of two strictly positive values. The normal distribution used for the prior of *rcd* is centered around 0, which amounts to a prior distribution around 1 and means an equal cell density. The normal distribution used for the prior of *rG2* is centered around -3 from prior knowledge that only a small proportion *Spirodela polyrhiza* cells are in G2 (this prior distribution samples rG2 values with a 95% likelihood from [0.001, 0.731] centered around 0.05).

Model 2:

$$dm Normal\left( \mu,\theta\right)$$

$$\mu=f_{dry}*m$$

We used following priors

$$f_{dry} e^{Normal\left( 0,2 \right)}$$

$$\theta Cauchy\left( 0,0.5 \right)$$

Supplementary Figures


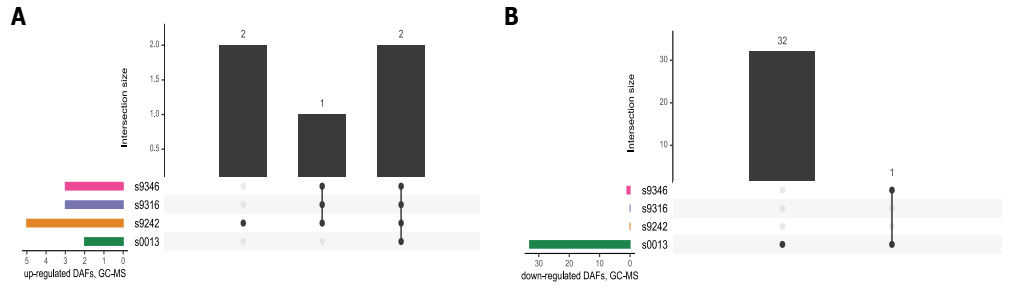


**Supplementary Figure 1.** UpSet plots of differentially abundant features (DAFs) in four *Spirodela polyrhiza* strains detected using GC-MS. Panel A displays features with significantly higher abundances in the tetraploids. Panels B represents the features with significantly lower abundances in the tetraploids.


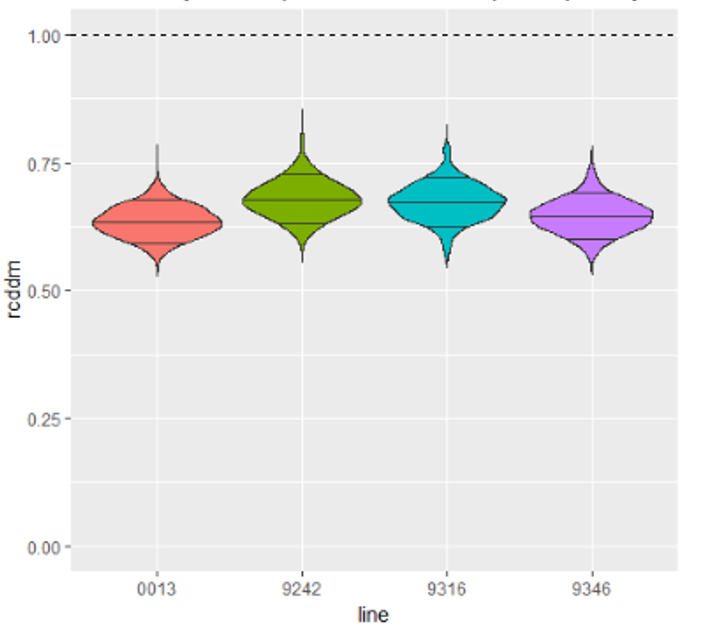


**Supplementary Figure 2.** Posterior distributions of strain-specific relative cell density (rcd) standardized for fresh mass (rcdfm) with 0.09, 0.5 and 0.91 percentiles indicated. The striped line indicates the value if tetraploid tissue had equal cell density compared to diploids. We estimated a relative cell density between 0.5 and 0.6 for all strains, which means that tetraploid strains all had only slightly over half number of cells compared to tissue of a similar fresh mass of their diploid counterpart.


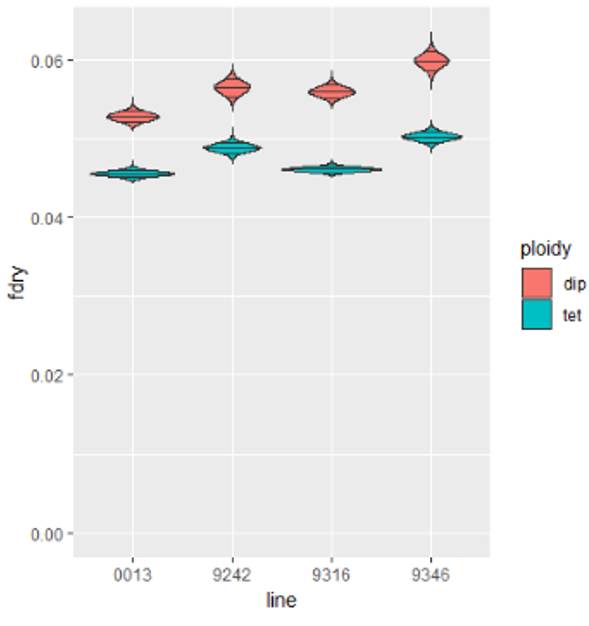


**Supplementary Figure 3.** Posterior distributions of strain-specific dry mass proportion of fresh mass, i.e., conversion factor from fresh to dry mass (fdry) for diploids (red) and tetraploids (blue) with 0.09, 0.5 and 0.91 percentiles indicated.


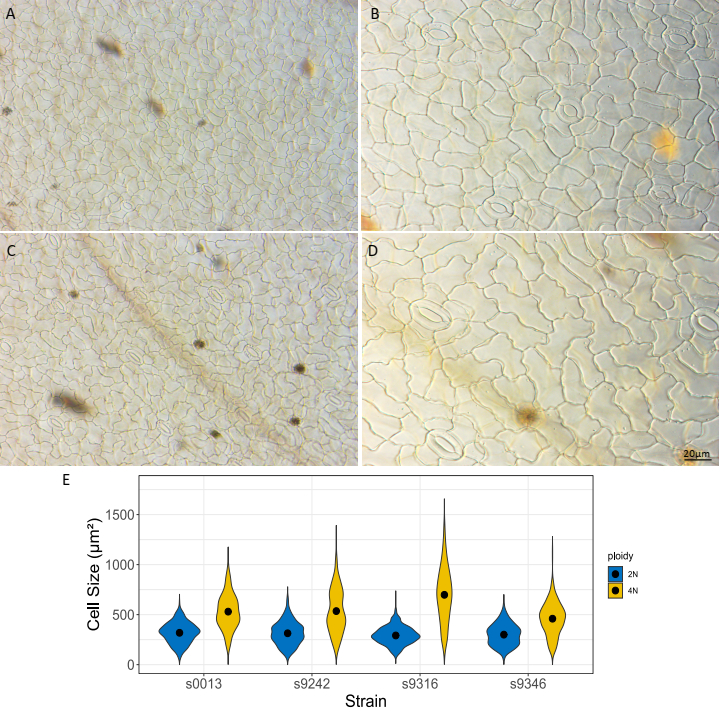


**Supplementary Figure 4.** The impact of WGD on the size of epidermal pavement cells of *Spirodela polyrhiza*. Micrographs (A-D) of the epidermis of diploid (A, B) and autotetraploid (C, D) plants at a magnification of 200× (A, C) and 400× (B, D). The distributions of the surface areas of the epidermal pavement cells from our different *Spirodela polyrhiza* strains (E). Data from diploid strains are depicted in blue, and data from tetraploid strains are shown in orange.
